# Sciatic nerve regeneration in rats by a promising electrospun collagen/poly(ε-caprolactone) nerve conduit with tailored degradation rate

**DOI:** 10.1186/1471-2202-12-68

**Published:** 2011-07-15

**Authors:** Wenwen Yu, Wen Zhao, Chao Zhu, Xiuli Zhang, Dongxia Ye, Wenjie Zhang, Yong Zhou, Xinquan Jiang, Zhiyuan Zhang

**Affiliations:** 1 Department of Oral and Maxillofacial Surgery, Ninth People's Hospital, Shanghai Jiao Tong University School of Medicine, Shanghai 200011, China; 2 Department of Stomatology, Provincial Hospital Affiliated to Shandong University, Jinan, Shandong, 250021, China; 3 Oral Bioengineering Lab, Shanghai Research Institute of Stomatology, Ninth People's Hospital, Shanghai Jiao Tong University School of Medicine, Shanghai Key Laboratory of Stomatology, Shanghai 200011, China

## Abstract

**Background:**

To cope with the limitations faced by autograft acquisitions particularly for multiple nerve injuries, artificial nerve conduit has been introduced by researchers as a substitute for autologous nerve graft for the easy specification and availability for mass production. In order to best mimic the structures and components of autologous nerve, great efforts have been made to improve the designation of nerve conduits either from materials or fabrication techniques. Electrospinning is an easy and versatile technique that has recently been used to fabricate fibrous tissue-engineered scaffolds which have great similarity to the extracellular matrix on fiber structure.

**Results:**

In this study we fabricated a collagen/poly(ε-caprolactone) (collagen/PCL) fibrous scaffold by electrospinning and explored its application as nerve guide substrate or conduit *in vitro *and *in vivo*. Material characterizations showed this electrospun composite material which was made of submicron fibers possessed good hydrophilicity and flexibility. *In vitro *study indicated electrospun collagen/PCL fibrous meshes promoted Schwann cell adhesion, elongation and proliferation. *In vivo *test showed electrospun collagen/PCL porous nerve conduits successfully supported nerve regeneration through an 8 mm sciatic nerve gap in adult rats, achieving similar electrophysiological and muscle reinnervation results as autografts. Although regenerated nerve fibers were still in a pre-mature stage 4 months postoperatively, the implanted collagen/PCL nerve conduits facilitated more axons regenerating through the conduit lumen and gradually degraded which well matched the nerve regeneration rate.

**Conclusions:**

All the results demonstrated this collagen/PCL nerve conduit with tailored degradation rate fabricated by electrospinning could be an efficient alternative to autograft for peripheral nerve regeneration research. Due to its advantage of high surface area for cell attachment, it is believed that this electrospun nerve conduit could find more application in cell therapy for nerve regeneration in future, to further improve functional regeneration outcome especially for longer nerve defect restoration.

## Background

For peripheral nerve repair, nerve autografts have always been considered as the "gold standard" for the restoration of structural and functional nerve regeneration. Yet autograft acquisitions are also faced with several limitations, such as the sensation loss of donor area, dimension discrepancies between donor and recipient nerves and most importantly, the lack of enough nerve sources for multiple nerve injuries. To seek alternatives for autografts, artificial nerve guides or nerve conduits have been introduced for the easy specification of conduit sizes and the availability for mass production [1,2].

For artificial nerve conduits, great efforts have been made directed by the aim to best mimic the structures and components of autologous nerve. With the progress of fabricating techniques during the previous decades, structures of nerve conduits have been greatly improved to satisfy different kinds of requirements including porous and fibrous channel wall with good permeability and degradability, along with proper mechanical properties to resist collapse when applied *in vivo*. Electrospinning is one of such prominent techniques which has been used to fabricate fibrous scaffolds for various regenerative medicine applications such as vascular reconstructions [3,4] and musculoskeletal tissue engineering [5-7]. Apart from their superior mechanical and physical properties, fibrous scaffolds made by electrospinning are recognized to be able to offer high surface area for cell attachment and possibly topographical signals for directing cellular functions due to their similarity to extracellular matrix structures [8]. This unique characteristic on structure promptly promotes wide exploration on its use for neural tissue engineering [9-12].

In terms of nerve conduit fabrication, material selection and optimization is also important for an ultimately good restoration outcome. By far materials used have been switched from silicone tubes at an earlier time to various degradable polymers. They can vary from natural purified extracellular matrix (ECM) components such as collagen and fibronectin to synthetic polymers such as polyglycolic acid (PGA) and poly(ε-caprolactone) (PCL) [1], among which collagen type I, PGA and poly-DL-lactide-caprolactone (PLCL) have been approved by U.S. Food and Drug Administration (FDA) and Conformit Europe (CE) to produce commercial conduits for use in clinical settings [13]. The technique of electrospinning is applicable to a wide variety of macromolecules ranging from natural biopolymers such as collagen, polysaccharides and silk to synthetic polymers such as poly(lactic acid) (PLA), poly(lactide-co-glycolide) (PLGA) and PCL or a blend of natural and synthetic materials, which provides wider selections for researchers to fully utilize the advantages of different materials [2].

Thanks to the unique fiber structure offered by scaffolds and wide material selection options of this technique, electrospinning recently have been valued by neurologists on fibrous scaffold fabrication for nerve regeneration research. However, until now most of these previous reports mainly focused on the interactions of electrospun fibers with neuronal and glial cells *in vitro*. To further testify the advantages of this new kind of scaffold as nerve conduits a large number of explorations are still needed.

**Figure 1:**
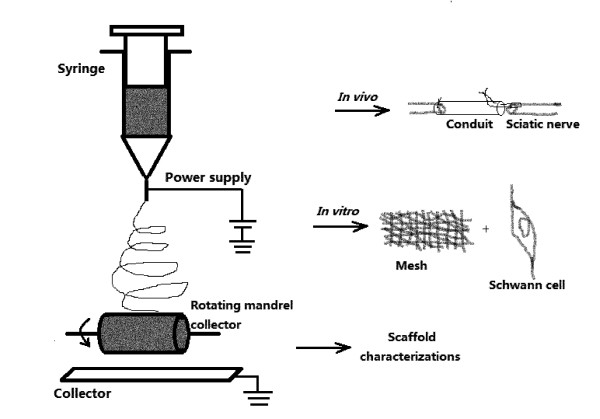
**Schematic illustration of the electrospinning setup and three major components of the experiment**. Electrospun fibers were collected onto a flat plate collector to form meshes, and collected onto a rotating mandrel to form guidance conduits. After scaffold characterizations, the electrospun collagen/PCL meshes were tested with Schwann cells *in vitro *and guidance conduits were sutured to connect two stumps of a rat sciatic nerve lesion *in vivo *(the suture was performed as shown in the figure).

As illustrated above, collagen and PCL are both biomaterials approved by FDA and CE and have gained widespread acceptance in clinical applications. Moreover, it is demonstrated that composite fibers produced by electrospinning of a blend of natural and synthetic macromolecules could combine the superior mechanical properties of synthetic polymers and the biocompatibility of natural polymers [14]. Based on these considerations, we designed and fabricated an electrospun collagen and PCL composite scaffold with good permeability and flexibility, and further assessed its biocompatibility *in vitro *with Schwann cells as well as its application as nerve conduits for rat sciatic nerve regeneration (Figure 1).

## Results

### Fabrication and characterizations of electrospun fibrous scaffolds

**Figure 2:**
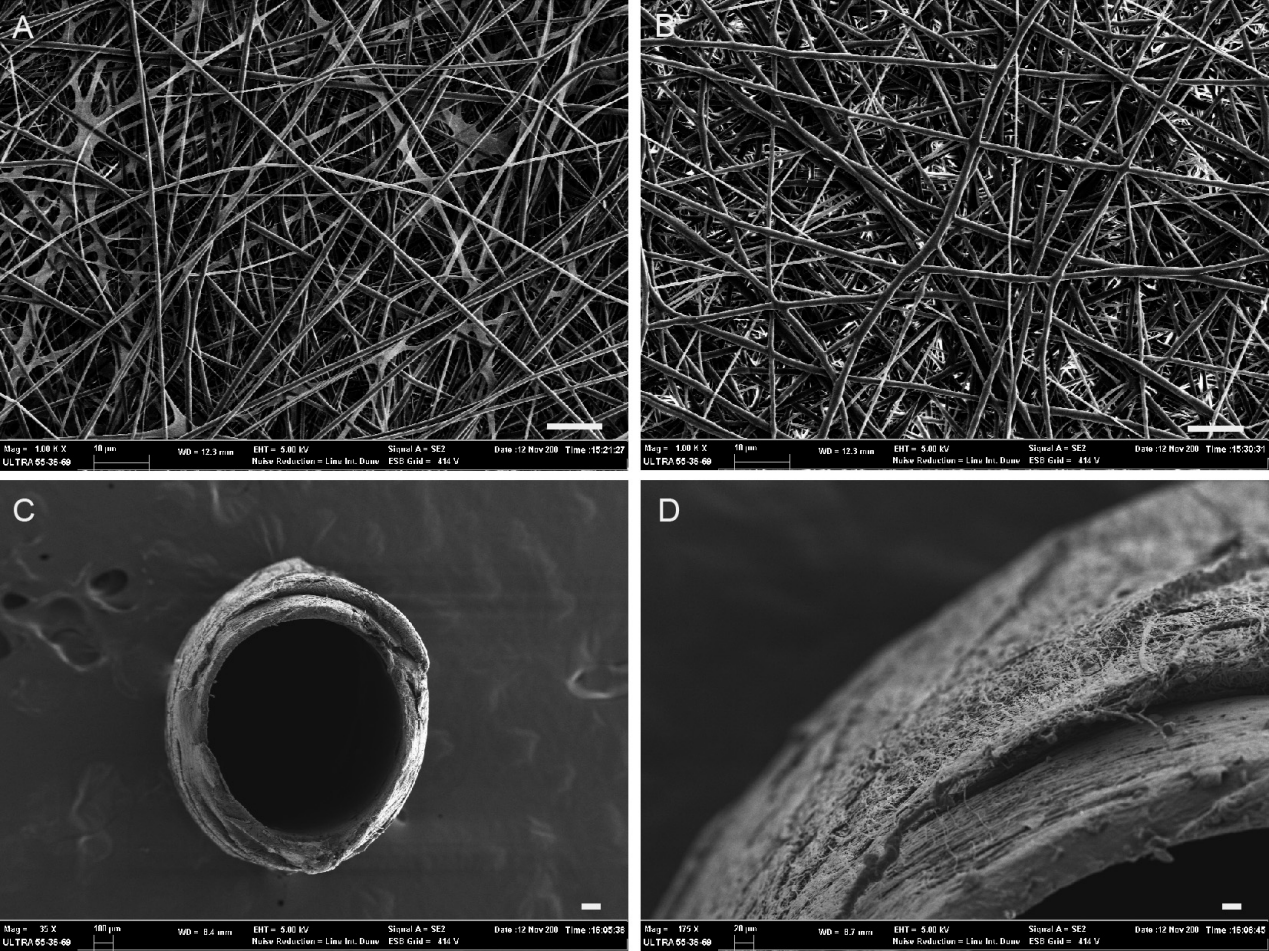
**SEM observations of electrospun fibrous meshes and guidance conduits**. A, collagen/PCL fibrous mesh. B, PCL fibrous mesh. C, electrospun collagen/PCL nerve conduit. D, the detailed outer wall morphology of collagen/PCL nerve conduit. (Bar in A,B = 10 μm; bar in C = 100 μm; bar in D = 20 μm).

Collagen/PCL blend solution and pure PCL solution were separately electrospun onto a flat plate to form randomly aligned fiber meshes by the same electrospinning parameters during this study. SEM images showed bead-free and relatively uniform fiber morphologies (Figure 2A,B). The diameters of collagen/PCL and PCL fibers were 675 ± 386 nm and 797 ± 330 nm respectively (Table 1). Electrospun collagen/PCL fibers which were collected onto a 1.2 mm diameter rotating mandrel formed a hollow guidance structure, as shown in Figure 2(C,D). Guidance conduits had uniform wall thickness of about 100-120 μm and inner diameter of 1.2 mm. They were formed by layers of fibers and pores among fibers (< 10 μm) may facilitate the exchange of nutrients and metabolic products between inside and outside of the conduit lumen.

**Table 1:** Average fiber diameter, hydrophilicity and mechanical properties of electrospun PCL and collagen/PCL fibrous meshes

Electrospun fibrous meshes (n = 3)	Fiber diameter (nm)	Water contact angle (°)	Tensile properties		
			
			Tensile strength (MPa)	Elongation at break (%)	Young's modulus (MPa)
PCL	797 ± 330	133.5 ± 0.9	4.36 ± 0.56	516.89 ± 43.63	2.02 ± 0.38
Collagen/PCL	675 ± 386	0	3.32 ± 0.09	34.54 ± 17.44	32.59 ± 1.25

Water contact angle measurement revealed the average contact angle values of electrospun PCL meshes were 133.5 ± 0.9 while those of collagen/PCL fibrous meshes presented as 0 (Table 1). Results showed that when combined with collagen, fibrous meshes greatly increase the hydrophilicity.

Mechanical testing results were also shown in Table 1 indicating differences between collagen/PCL and PCL fibrous meshes in tensile properties. The ultimate tensile strength of collagen/PCL meshes (3.32 ± 0.09) was smaller than that of PCL (4.36 ± 0.56) (p = 0.033), as well as results of elongation at break (p < 0.01). But the collagen/PCL composite material had greater elasticity (32.59 ± 1.25), that is Young's modulus, compared to that of pure PCL (2.02 ± 0.38), which indicated collagen/PCL material could better stand force stretching and be more suitable for *in vivo *use under complicated conditions (p < 0.01).

The above results agreed with our expectations that composite electrospun material possessed superior hydrophilic and elastic properties which might be a more suitable substrate for subsequent *in vitro *and *in vivo *experimental explorations than pure one single synthetic polymer.

### *In Vitro *biocompatibility

#### Acquisition and characterization of rat Schwann cell

**Figure 3:**
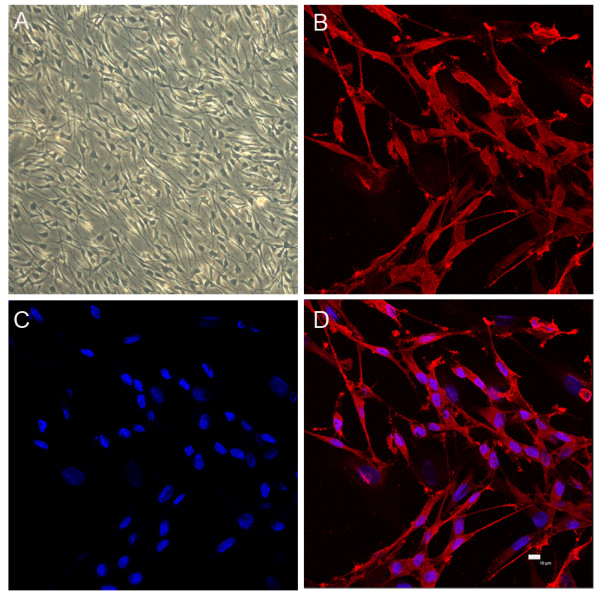
**Schwann cell culture and characterization of p75^NTR ^**. After two rounds of purification, postnatal rat Schwann cells at the third passage presented a phase-refractile, bipolar or tripolar cell character, with a growth tendency to connect with each other. A, light microscopy of Schwann cells (× 100 magnification). B, anti- p75^NTR ^staining. C, nuclei DAPI staining. D, image merged from B and C (Bar = 10 μm).

According to a new easy and efficient method to obtain Schwann cells from animal sciatic nerves reported recently [15,16], purified Schwann cells were successfully achieved by using differential detachment reactions to complex collagenase. Figure 3A showed postnatal rat Schwann cells after two rounds of purification. They presented a phase-refractile, bipolar or tripolar cell character with a growth tendency to connect with each other. Immunocytochemistry proved that they were all stained positively for p75^NTR^, a cell surface molecule used for Schwann cell characterization and selection [17,18] (Figure 3B-D).

#### Cell morphology and proliferation on electrospun collagen/PCL meshes

**Figure 4:**
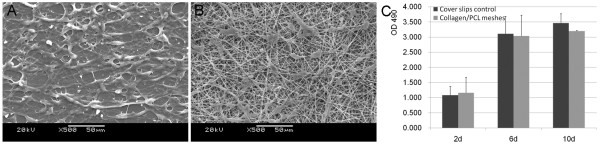
**SEM images of Schwann cell morphology on materials and the proliferation assay**. A, on glass cover slips (control). B, on electrospun collagen/PCL fibrous meshes 3 days after cell seeding. Cell proliferation by MTS assay was shown in (C). There were no statistically significant differences between two groups at 2,6,10 days point (p > 0.05).

SEM observations showed Schwann cells adhered and spread well along fibers after seeded on electrospun collagen/PCL fibrous meshes for 3 days (Figure 4B). Cells on the surfaces of meshes presented a little fewer than those grown on cover slips (Figure 4A). Quantitative data of Schwann cell proliferation on cover slips and electrospun collagen/PCL meshes at 2,6,10 days point were demonstrated by MTS assay (Figure 4C). No statistically significant differences were found between two groups at the above time points (p > 0.05).

### *In Vivo *experiment

#### Animals and surgical outcomes

**Figure 5:**
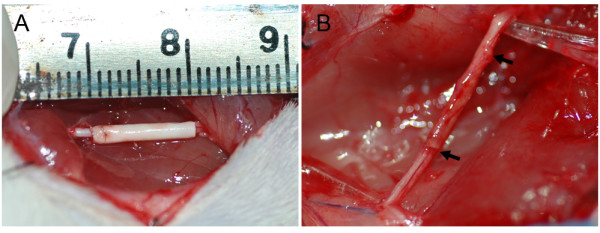
**Experimental model**. Surgical implantation of a collagen/PCL nerve conduit (collagen/PCL NC) bridging an 8 mm sciatic nerve defect in rats (A) and the view of the regenerated nerve 4 months postoperatively (B). Electrospun artificial nerve conduit implanted in rats after 4 months was surrounded by a thin layer of fibrous connective tissue with abundant capillaries. The original boundary areas between conduit and nerve (black arrows) became blurry and the conduit degraded gradually as regenerated nerve went through.

At 4 months after implantation surgery, animals of both autograft and collagen/PCL NCs groups were found to have recovered from foot ulcers on the left side with minor visible gastrocnemius muscle degeneration (n = 6 respectively), while animals of non-grafted group suffered from distinctive muscle atrophy on the left side (n = 3). In autograft group, implanted nerves fused completely with proximal and distal nerve stump tissues. In collagen/PCL NCs group, conduits were surrounded by a thin layer of fibrous tissue abundant in capillaries. There was no apparent neuroma formation or serious chronic inflammatory reaction. The original boundary areas between conduit and nerve became blurry as the regenerated nerve grew into the conduit and the conduit almost degraded, losing their original guidance structures at the observation endpoint (Figure 5). In non-grafted group, no regenerated nerves between proximal and distal nerve stumps were found.

#### Electrophysiological assessment

**Figure 6:**
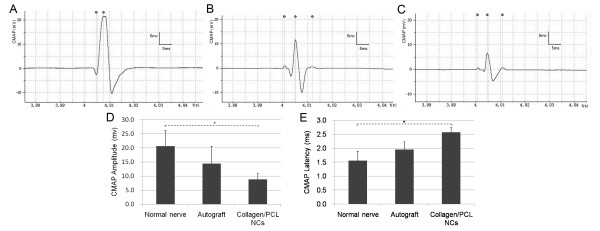
**Evoked compound muscle action potential (CMAP) detection of gastrocnemius muscle 4 months postoperatively**. Representative data of the unoperated normal nerve group (A), the autograft group (B) and collagen/PCL NCs group (C). No signals were recorded and no muscle contractions were observed in non-grafted group. Data comparison of CMAP amplitude and latency was shown in (D) and (E) (n = 6). There were no statistically significant differences between autograft group and collagen/PCL NCs group, or between autograft group and normal nerve group (p > 0.05). Significance existed between normal nerve group and collagen/PCL NCs group (*p < 0.05).

Electrophysiological studies were performed to evaluate functional target reinnervation of regenerated nerves through nerve grafts. CMAP was evoked and recorded followed by measurements of amplitude and latency of signals. Representative signal data were shown in Figure 6. For amplitude measurements, there were no statistically significant differences between autograft and collagen/PCL NCs groups, or between autograft and normal nerve groups (p > 0.05). Significance existed between normal nerve and collagen/PCL NCs groups (*p < 0.05). The same statistical results were found in the analyses of latency. No signals were recorded and no muscle contractions were observed in non-grafted group. Results indicated collagen/PCL NCs group reached similar level of muscle reinnervation as autograft, but still was in a pre-mature stage to be improved towards normal nerve functions.

#### Nerve histology and immunohistochemistry

**Figure 7:**
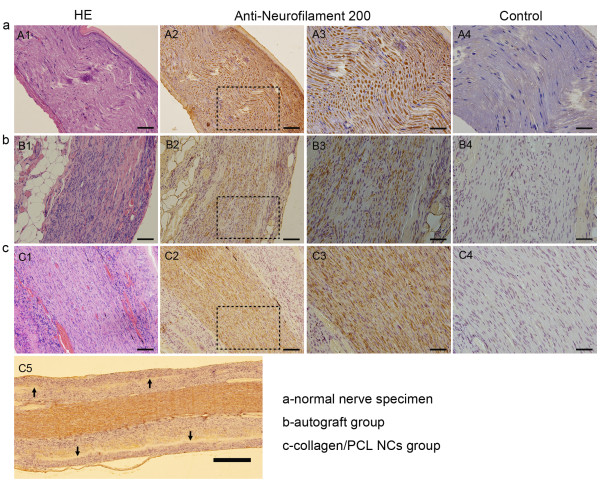
**Longitudinal histology of regenerated nerves for HE staining and anti-neurofilament staining under light microscopy**. a, normal nerve specimen. b, midpoint view of regenerated nerve in autograft group. c, midpoint view of regenerated nerve in collagen/PCL NCs group. A1,B1,C1, HE staining; A2-A4,B2-B4,C2-C5, anti-neurofilament staining; A3,B3,C3, magnified views of the square area in A2,B2,C2, respectively. A4,B4,C4, corresponding specimens as blank controls without primary antibody incubation. C5, overview image of the mid-portion of regenerated nerve in collagen/PCL NCs at 4 × magnification. Regenerated nerve fibers in autograft and collagen/PCL NCs groups were positively stained with neurofilament 200 similarly as normal nerve. Electrospun nerve conduits almost degraded and only a few of residual materials (black arrows) could be found inside the wall area 4 months postoperatively (C5). (Bar in A1,A2,B1,B2,C1,C2 = 100 μm; bar in A3,A4,B3,B4,C3,C4 = 50 μm; bar in C5 = 500 μm).

In HE staining, normal nerve presented morphologies of regular nerve fiber alignment and sparse oval Schwann cell nuclei among fibers (Figure 7A1). In autograft group (Figure 7B1), regenerated pre-mature nerve fibers were surrounded by epineurium consisting of fibrous connective and adipose tissue. Among nerve fibers a large number of Schwann cell nuclei and a few collagen fibers could be seen. In collagen/PCL NCs group (Figure 7C1), inside the conduit lumen regenerated nerve fibers aligned in comparatively regular way and abundant Schwann cell nuclei still presented. Different from autograft group, there were fewer collagen fibers grown into regenerated nerves in this group, which might be due to the protection of conduit walls. With anti-neurofilament 200 staining, fibers regenerated through the conduit lumen were positively stained similarly as normal nerve and autograft nerve (Figure 7A2,B2,C2). Electrospun nerve conduits degraded as regenerated nerves went through and only a few of residual materials could be found inside the wall area 4 months postoperatively (Figure 7C5).

#### Toluidine blue staining and transmission electron microscopy

**Figure 8:**
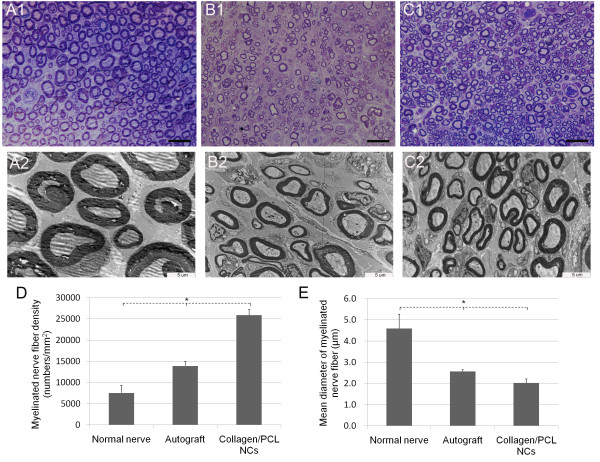
**Histologic observation and morphometric analysis of regenerated nerves on transverse sections**. Light microscopy observation of toluidine blue staining of transversely semithin nerve sections (A1-C1) and transmission electron microscopy of ultrathin nerve sections (A2-C2). A1,A2-normal nerve group as control. B1,B2-autograft group. C1,C2-collagen/PCL NCs group. Normal sciatic nerve (A1, A2) had myelinated nerve fibers with comparatively uniform and large diameter and thick myelin sheath. Regenerated nerves (B1,B2,C1,C2) either from autograft group or collagen/PCL NCs group still presented immature morphologies 4 months postoperatively. Quantitative analysis of myelinated nerve fiber densities (D) showed differences among normal nerve, autograft and collagen/PCL NCs groups and between every two groups were all statistically significant (*p < 0.05), as well as the result analysis of mean inner diameter (axonal diameter) of myelinated nerve fibers showed in E. (Bar in A1,B1,C1 = 20 μm; bar in A2,B2,C2 = 5 μm).

As shown in Figure 8, normal sciatic nerve had dense myelinated nerve fibers with comparatively uniform size and large diameter. Axons in myelinated nerve fibers were nearly all surrounded by myelin sheath with even thickness. High magnifications of midpoint view of regenerated nerves 4 months postoperatively either from autograft or collagen/PCL NCs group presented immature morphologies - nerve fiber diameters had a wide distribution range, but usually smaller than mature myelinated ones; fibers of small diameter were fairly much more than normal nerve; myelin sheath of axons were thinner and more Schwann cells could be seen. Quantitative analysis of myelinated nerve fiber densities could further confirm the above findings. Differences among normal nerve, autograft and collagen/PCL NCs groups and between every two groups were all statistically significant (p < 0.05), as well as the result analysis of mean inner diameter (axonal diameter) of myelinated nerve fibers showed in Figure 8E.

#### Gastrocnemius muscle histology and muscle weight ratio

**Figure 9:**
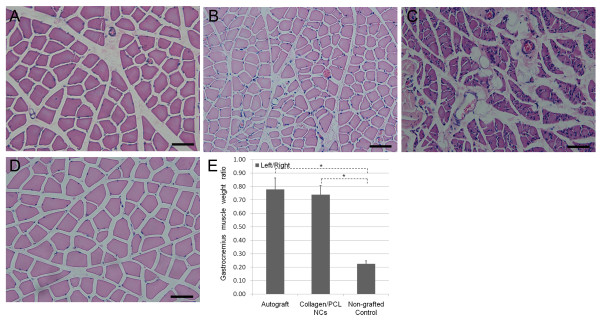
**Gastrocnemius muscle histology and analysis results**. Histology observation of gastrocnemius muscle by cross-sectional HE staining (A-D) and quantitative analysis of bilateral gastrocnemius muscle weight ratio (E). A, autograft group; B, collagen/PCL NCs group; C, non-grafted group; D, unoperated muscle in normal nerve group. Compared to normal muscle morphology (D), gastrocnemius muscles suffering from degeneration presented with smaller muscle cell sectional area, more fibrous connective tissues and blood vessels. In autograft group(A) and collagen/PCL NCs group (B), muscle atrophy after nerve injury was rescued to a great extent while muscles in non-grafted group (C) heavily degenerated. Quantitative analysis of left (experimental side)/right (unoperated side) muscle weight ratio showed similar tendency of atrophy changes. The muscle weight ratio of animals in normal nerve group was defined as 1. *p < 0.05 versus non-grafted group. No significant difference found between autograft group and collagen/PCL NCs group (p > 0.05).

As Figure 9 indicated, normal gastrocnemius muscle had larger muscle cell sectional area, and fewer fibrous connective tissues among muscle bundles. However, muscles suffering from nerve injury and atrophy changes presented smaller muscle cell sectional area, as well as more fibrous connective tissues and blood vessels. In autograft and collagen/PCL NCs groups, muscle atrophy after nerve injury was rescued to a great extent while muscles in non-grafted group heavily degenerated. Analysis of left (experimental side)/right (unoperated side) muscle weight ratio also reflected the above tendency of atrophy. Ratio comparisons of autograft or collagen/PCL NCs group to non-grafted group were statistically significant (p < 0.05). Interestingly, there were no statistically significant differences between autograft and collagen/PCL NCs groups (p > 0.05), revealing electrospun nerve conduits could have similar effects on preventing muscle atrophy as autograft.

## Discussion

In this study, we have successfully fabricated a collagen/PCL composite fibrous material by electrospinning and explored its application as nerve guide substrate or conduit *in vitro *and *in vivo*.

As a simple and versatile fabrication technique, electrospinning is a good choice to make fibrous scaffolds such as nerve conduits with tailored porosity and degradation rate, which can easily provide mass production of different-sized conduits made of nanofibers or submicron fibers [2,14]. So far diameter and morphology of electrospun fibers are controlled mostly empirically, relying on variation of solution concentration, molecular weight of the polymer and ratios of different polymers for composite material electrospinning [8]. Based on these principles and our previous explorations, we tried parameters of total solution concentration (10%) and collagen/PCL weight ratio (1:1) in this study, and finally successfully produced fibrous meshes and porous nerve conduits with fibers of 675 ± 386 nm in diameter.

As is known, PCL is one kind of aliphatic and degradable polyesters with good tensile properties and slow degradation kinetics. But its bioinert and hydrophobic properties limit its use as a biomedical material [19]. Collagen as the main structural protein of ECM possesses excellent biocompatibility. Yet as a biomedical material for tissue engineering, it often requires additional chemical treatments of cross-linking to decrease its degradation rate for long-term *in vivo *use, which could have negative effects on its bioactivity [20]. So a combination of collagen and PCL properly may compensate their shortcomings when applied for tissue-engineering. Our results showed that compared to pure PCL fibers, collagen/PCL fibers possessed better hydrophilicity and flexibility. Due to the addition of collagen, water contact angle of collagen/PCL meshes dramatically decreased which could facilitate cells to attach and spread. Meanwhile, PCL improved scaffold's load-bearing abilities, and together with collagen made this composite material possess greater Young's modulus and slowed its degradation rate without additional treatments. Good flexibility and proper degradation rate were also important factors for a tissue-engineered conduit to resist rearing and stretching forces and retain stable shape during nerve regeneration process [21]. If nerve conduits had large rigidity, it might exert chronic compression to regenerated nerves. If too soft without enough mechanical support, it is impossible to be manipulated in an implant surgery and the conduits could not bear forces and collapse *in vivo*. Panseri S et al. found their electrospun PLGA/PCL tubular conduits collapsed in 40% of the treated rats for a 10 mm sciatic nerve gap repair [12]. Therefore it is essential to explore the details on mechanical characteristics of nerve conduits. Our mechanical testing data and animal experiment results showed that the combination of collagen and PCL had good flexibility and gave good performance in resisting collapse and stretch forces *in vivo *which might provide some detail evidence for deepening our mechanical understanding to synthetic nerve conduits.

Different from central nervous system, peripheral nerves have some kind of capacity to regenerate after injuries. In this process, Schwann cell, the dominating glial cell type in peripheral nervous system plays crucial roles. They undergo changes of dedifferentiation and proliferation, secret neurotrophic factors and extracellular matrices and surround regenerating axons to form myelin sheaths which all contribute to a favorable environment for nerve regeneration [1]. So before applying artificial nerve conduits *in vivo*, we first tested the biocompatibility of electrospun collagen/PCL fibrous material with Schwann cells through adhesion observation and proliferation assays *in vitro*. Previous research showed that Schwann cells well adhered on randomly aligned electrospun fibers, stretched across multiple fibers and elongated along the fiber axes [22]. Our SEM observations were consistent with this finding. For proliferation assays, we found that postnatal Schwann cells could proliferate well just as on cover slips when seeded on electrospun fibers at the three observation time points. The above tests suggested that electrospun collagen/PCL composite material could be used as a good substrate for cell attachment and proliferation which agreed with previous results on the interactions of electrospun collagen/PCL fibers with glial cells [9,23].

For *in vivo *test in peripheral nerve regeneration studies, sciatic nerve model is most commonly used for its adequate length and space at the mid-thigh for experimenters to manipulate a surgery and implant a graft easily [24]. In our experiment, we used adult F344 rats to test the efficacy of electrospun collagen/PCL porous nerve conduits in bridging an 8 mm sciatic nerve defect gap. To evaluate axonal outgrowth and muscle reinnervation, a combination of traditional methods was used, including electrophysiology and histomorphometry.

For electrophysiological assessment, the amplitude of CMAP is one commonly used parameter which indirectly reflects the numbers of regenerated motor nerve fibers and the extent of muscle reinnervation while latency is an indirect parameter which refers to maturation of nerve fibers [25]. Results of amplitude and latency measurements of CMAP in this experiment indicated collagen/PCL NCs group achieved similar functional muscle reinnervation as autografts. Besides, gastrocnemius muscle weight ratio is another parameter which could also conveniently give information about the efficacy of reinnervation by evaluating the extent of muscle atrophy in sciatic nerve lesion model [26]. It is discovered that the maintenance of muscle mass is controlled by a balance between protein synthesis and protein degradation pathways. When a muscle is denervated as a consequence of nerve injury, the balance is destroyed and shift to degradation tendency which leads to decreased muscle cell size, muscle weight loss and hyperplasia of connective tissues [27]. In this study compared to non-grafted conditions, electrospun collagen/PCL NCs successfully prevented serious muscle weight loss as autografts did either from muscle weight ratio comparisons or histological observations, which further necessitated nerve guidance structures in promoting nerve regeneration. It should be noted that although HE staining on paraffin sections is more susceptible to technical artifacts than on frozen ones, an apparent tendency of muscle atrophy prevention in collagen/PCL NCs group and autograft group can be clearly observed from their histological morphologies when compared to non-grafted group. Similar histological method and results could be found in related research [28,29].

In nerve histology examination, regenerated nerves from autograft and collagen/PCL NCs groups all successfully grew through the gap and connected the proximal and distal nerve stumps. However, transmission electron microscopy and quantitative analyses of myelinated nerve fiber density and axonal diameter showed that those regenerated nerve fibers were still in a pre-mature stage of myelin sheath formation and maturation. Our experimental results of larger fiber density but relatively smaller-sized regenerated fibers in collagen/PCL NCs group might give evidence for the pruning hypothesis proposed by Brushart TM [30]. After injury, proximal nerve stumps generated multiple collateral sprouts and over time nerve fibers failing to establish connections with target muscles or incorrectly projecting into the sensory branch would be pruned away [31]. Therefore, we believe that on condition that observation time prolonged, nerve fiber density in collagen/PCL NCs group would decrease and nerve fibers would gradually become uniformly larger-sized and mature ones [32]. In repair of nerve autograft, we speculate it is endoneurial tube that facilitates axonal outgrowth, which contributes to unnecessary sprouting following nerve injury, resulting in relatively fewer nerve fibers than in collagen/PCL NCs group.

Through a series of systematic sum and analysis researchers conclude that an appropriate combination of evaluation methods is preferred aiming at different research questions for different methods only illustrate discrete aspects of nerve regeneration [26,33]. Because our exact aim is mainly to illustrate the role of nerve conduit in enhancing nerve fiber regeneration and the change of conduit itself in this process, functional or behavioral tests were not conducted in this experiment. For functional tests, walking track analysis is often adopted in rat sciatic nerve lesion model to assess motor functional recovery by calculating sciatic functional index (SFI) [26,34,35], while behavioral test such as Von-Frey test is used for sensory recovery measurement [12]. Recently video recordings of rat gait have been developed to analyze motor function recovery in similar studies [36]. These are all useful assessments to be applied in our future researches on functional recovery. Our examinations in this experiment demonstrated that although there were some morphological differences on regenerated nerve fibers, collagen/PCL NCs achieved similar electrophysiological and muscle histological results as autografts. As an artificial nerve conduit, electrospun collagen/PCL NC successfully protected regenerated nerves through the lesion defect from interruption of scar tissues, thus shortened time to earliest muscle reinnervation which was thought as the determinant in functional recovery [37].

Moreover, at the time of observation endpoint (4 months postoperatively), the implanted nerve conduits in our experiment almost degraded, losing their original guidance structures which well matched the nerve regeneration rate. Degradation rate is emphasized as an important design consideration for artificial nerve conduits because non-degradable tubes would compress regenerated nerves in the long term. Studies of functional recovery of transected peripheral nerves have indicated that there is a critical time period (the first 10-12 weeks following injury) for the regenerating axons to grow through the distal nerve stump for optimal recovery to occur [37,38]. During this time period a relatively "undisturbed" microenvironment is required and afterwards a tendency of nerve conduit degradation is preferred. *In vitro *degradation test of our electrospun collagen/PCL scaffold showed that after 3-month immersion in PBS at a constant temperature of 37°C an approximate amount of 81.7% weight was lost as the scaffold degraded and became amorphous substances (data was not shown). Based on predecessors' work and our preliminary results, we finally chose a time point of 4 month to observe the conditions of nerve regeneration and conduit degradation *in vivo*.

Our macroscopic and microscopic results proved that the electrospun collagen/PCL nerve conduit with a wall thickness of 100-120 μm facilitated more axons regenerating through the gap and almost degraded at the observation endpoint of 4 months after injury (a time point shortly after the critical stage of axonal regeneration). Thus, we conclude that the degradation rate of electrospun collagen/PCL nerve conduits is well tailored to the need of a relatively "undisturbed" microenvironment for favorable peripheral nerve regeneration. The advantages of electrospun collagen/PCL nerve conduits on mechanical shape retaining and degradation rate controlling might give some useful supplements to previously published work, in particular those related researches on electrospun tubes in this field [12].

## Conclusions

Here, we demonstrate a collagen/PCL porous nerve conduit fabricated by electrospinning could support favorable rat sciatic nerve regeneration with the advantages of high surface area for cell attachment and tailored degradation rate. Based on this, our future studies will attempt the incorporation of luminal aligned electrospun fibers encapsulating growth factors or even cells via cell electrospinning [39], as well as the addition of electrical materials to obtain preferred regeneration outcome, especially for longer nerve defect restoration.

## Methods

### Materials

Porcine collagen type I(M_w _= 80,000-100,000) was purchased from Mingrang Products Company (Sichuan, China). Poly(ε-caprolactone) (PCL, M_n _= 70,000-90,000) was purchased from Sigma-Aldrich (St. Louis, MO, USA). 1,1,1,3,3,3-Hexafluoro-2-propanol (HFP) was purchased from Merck (Hohenbrune, Germany). Chloroform, methanol and other chemicals were common laboratory analytical reagents from China. All chemicals were used as received unless stated otherwise.

### Electrospinning

Electrospun collagen/PCL fibers were fabricated using a blend of collagen and PCL with a weight ratio of 1:1. Both collagen and PCL were dissolved in HFP at a total concentration of 10% (w/v) and stirred overnight. Control PCL solution for electropinning was formed when PCL was dissolved in chloroform/methanol (75:25 v/v) with the same concentration of 10% (w/v). Briefly, solutions were electrospun using a high voltage of 16 kV between the solution and the receiving apparatus with a constant distance of 12 cm. Solutions were delivered with a 10 ml polypropylene syringe through a 0.9 mm tip needle at a flowing rate of 2 ml/h using a syringe pump of medical use. Fibers were collected onto a flat plate coating a layer of aluminum foil to form meshes, and collected onto a die steel mandrel of 1.2 mm in diameter with a rotating rate of 500-600 rpm to form guidance conduits. All electrospun fibers were stored under vacuum for 48 h to remove residue solvents.

Additionally, for cell culture studies, electrospun collagen/PCL fibers were collected onto round cover slips placed on the surface of aluminum foil. After vacuum drying cover slips were carefully cut out along the round contour from the meshes. The glass cover slips with a diameter of 12 mm were purchased from Electron Microscopy Sciences (PA, USA) which were pre-treated by the manufacturer for neural cell culture use.

Fibers collected onto mandrels were also carefully demolded to form hollow guidance structures. They were cut into segments of 1-1.2 cm ready for use *in vivo *as nerve conduits. All the formed materials either on cover slips or hollow guidance conduits were sterilized under an ultraviolet lamp for 2 h before biological use.

### Characterizations of electrospun fibrous scaffolds

#### Fiber morphologies

Fiber morphologies of electrospun fibrous meshes and guidance conduits were observed by scanning electron microscopy (SEM) (Model Ultra 55, ZEISS, Germany) at an accelerating voltage of 5 kV after sputter coating with gold. One hundred fibers from a randomly selected area per SEM image (n = 3) for each kind of material were measured and data of fiber diameters were obtained. Meanwhile, the wall thickness of guidance conduits was also measured.

#### Wettability Properties

Surface contact angle of electrospun fibrous meshes was examined using a contact angle measurement system (OCA-20, Dataphysics, Germany) and distilled water was used as the testing liquid. Six samples from each kind of material were tested at room temperature with each sample observed for 5 minutes and the average values and standard deviations were calculated.

#### Mechanical testing

Electrospun meshes prepared with uniform size (25 mm of length, 10 mm of width) (n = 3) were tested in the study. Thickness was measured by averaging three points of the sample using a micrometer caliper. Briefly, mechanical testing was performed on electrospun collagen/PCL and pure PCL fibrous meshes respectively, using a uniaxial load testing equipment (EZ20, Lloyd instruments, AMETEK, USA). The crosshead speed was set at 5 mm/min and the test was stopped when the load decreased to 1% after the onset of failure. Material test and data analysis software NEXYGEN (Lloyd instruments, AMETEK, USA) was used to manipulate the whole test and calculate tensile properties of tested materials, including parameters of tensile strength, elongation at break and Young's modulus.

### *In Vitro *biocompatibility

#### Rat Schwann cell culture and immunostaining characterization

Animals used in this experiment were all obtained from the Ninth People's Hospital Animal Center (Shanghai, China). All animal procedures were approved by Animal Experiment and Care Committee of Ninth People's Hospital affiliated to Shanghai Jiao Tong University School of Medicine. Briefly, postnatal F344 rat (3-4-day-old) sciatic nerve segments were harvested using microsurgical instruments aseptically. Tissue enzymatic digestion was performed in Collagenase NB 4 (0.2% w/v, Serva, Germany) at 37°C for 1 h in a shaker. Then nerve tissues were further disaggregated by pipetting for 2 min. After centrifugation at 1000 rpm for 10 min, the supernatant was discarded and the cell pellet was resuspended in Schwann cell culture medium (SCCM) that was composed of DMEM with 10% fetal bovine serum (FBS, Hyclone, Australia), 2 μM forskolin (Sigma, USA) and 10 ng/ml heregulin-β-1 (Pepro Tech, USA) [15,40]. Then cell suspension was plated onto 25-cm^2 ^flasks (Greiner Bio-one, Germany) which were coated with 400 ng/ml laminin (Sigma, USA) at a density of 2.0 × 10^4 ^cells/cm^2^. After 48-h culture, cells were digested by 0.05% Collagenase NB 4 at 37°C for 30-40 min with continual gentle shaking to release detaching cells which mainly were Schwann cells. This purification procedure aimed to get purer Schwann cells using differential detachment reactions, eliminating fibroblasts as much as possible [15].

To characterize Schwann cells obtained after two rounds of purification, cells seeded on cover slips were fixed with 4% paraformaldehyde at 4°C for 30 min, washed with phosphate buffered saline (PBS, pH = 7.2) for three times, permeated by 0.1% Triton for 10 min and blocked by 1% FBS for 30 min at room temperature. Afterwards, slips were incubated with rabbit anti-P75^NTR ^(polyclonal; Abcam, UK; 1:100 diluted in PBS) at room temperature for 2 h. Cells without primary antibody staining were used as a blank control. After three washes with PBS, slips were further incubated with Alexa Fluor^® ^546 goat anti-rabbit IgG (Invitrogen, USA; 1:1000 diluted in PBS) at room temperature for 1 h. Cell nuclei were stained with 4', 6-diamidino-2-phenylindole (DAPI). Labeled cells were visualized under a confocal laser scanning microscope (Leica, Germany).

#### Cell morphology and proliferation on electrospun collagen/PCL meshes

To investigate *in vitro *biocompatibility of this electrospun collagen/PCL material, fiber meshes collected on cover slips were used. They were placed in wells of 24-well plates (Greiner Bio-one, Germany), washes twice using PBS and immersed in DMEM overnight before cell seeding. Schwann cells after two rounds of purification were seeded on cover slips coated with electrospun collagen/PCL fibrous meshes at a density of 2.0 × 10^4 ^cells/cm^2 ^and cultured in SCCM. Cells seeded on blank cover slips were used as controls.

To observe Schwann cell spreading and adhesion morphology on electrospun collagen/PCL fibrous meshes, SEM was used. For preparation, cells grown on meshes for up to 3 days together with cover slips were fixed in 2% glutaraldehyde at room temperature for 2 h. Subsequently, samples were dehydrated through a series of graded ethanol solutions, dried in the air and then gold-sputtered. Cell morphology images on fiber meshes were obtained using a SEM (JEOL JSM-6380, Japan) at an accelerating voltage of 20 kV.

To assess cell proliferation on electrospun collagen/PCL fibrous meshes, MTS assay (Cell Titer 96 Aqueous Non-Radioactive Cell Proliferation Assay, Promega, Madison, WI, USA) was used after 2,6,10 days of cell seeding. Firstly, cover slips coated with and without fiber meshes were carefully moved from previous wells to new 24-well plates, respectively. A volume of 200 ul culture medium was added to each well, and then 40 ul solution taken from the mixture of MTS (20 ml) and PMS (1 ml) was added. Plates were returned to cell incubator and incubated for 3 h. The MTS formazan products accumulated in the cytoplasm of viable cells were soluble in culture medium and their blue dark color was proportional to the numbers and metabolic activities of tested cells. Finally, absorbance was measured at 490 nm using a microplate reader (EL × 800, BioTek, USA). Each group at every time point contains three samples and the test was conducted twice.

### *In Vivo *experiment

#### Animals and surgical procedures

Adult male F344 rats (200-250 g) were randomly allocated into four groups: normal nerve group (n = 6), nerve autograft group (n = 6), collagen/PCL nerve conduits group (collagen/PCL NCs) (n = 6) and a non-grafted group (n = 3). The normal nerve group served as positive control and the non-grafted group as negative control. Left sciatic nerve was chosen as the experimental side in each animal. All animals were anaesthetized by an intraperitoneal injection of 10% chloral hydrate (0.4 ml/100 g body weight). Then the sciatic nerve was exposed by making a skin incision and splitting the underlying muscles in the left thigh. In the normal nerve group, nerves were given no lesions or treatments. In the nerve autograft group, an 8 mm long piece of nerve was resected, then reversed 180°and re-implanted across the defect. In the collagen/PCL NCs group, a 5-6 mm long piece of nerve was removed, leaving an 8 mm defect gap between two nerve stumps following their retraction. Then, a 10 mm long guidance conduit was sutured to bridge the defect, connecting the proximal and distal nerve stumps with both ends entering the conduit by 1 mm. In the non-grafted group, there was no bridge to connect two stumps which were both ligated with sutures. All the implantation procedures were conducted under microscopes using microscopy instruments. Nerve-to-nerve and nerve-to-conduit connections were completed using 11-0 nylon sutures. Muscle and skin incisions were subsequently closed using 4-0 sutures. Rats after surgery were housed, fed routinely and monitored for changes in their ordinary conditions and motor activities.

#### Electrophysiological assessment

Four months after surgery, animals were anaesthetized using chloral hydrate again and the sciatic nerves were exposed. Adipose and fibrous tissues surrounding the sciatic nerve trunk were carefully removed. Electrical stimulation was applied to nerves by placing bipolar hooked silver stimulating electrodes proximal to the nerve graft or conduit. Stimulating mode was set as pulse mode (stimulus intensity 750 mv, frequency 1 Hz, duration 1 ms). And a pair of concentric needle electrodes was inserted in the belly of gastrocnemius muscle and connected to a dual bioelectric amplifier (AD instruments, Australia) to record the evoked compound muscle action potential (CMAP). Meanwhile, a recording and analysis system Powerlab was used to record signal waves and a software Chart was used to measure amplitude and latency of CMAPs acquired (amplitude was defined as the height by signal level minus the baseline at the peak; latency was defined as the time interval from the stimulus artifact to the start of the response). They were all from AD instruments. Normal nerve CMAP from animals without lesions was also measured using the same setup and testing method at room temperature.

#### Nerve and gastrocnemius muscle harvest

Immediately after electrophysiological assessment, animals were given an overdose intraperitoneal injection of anaesthetics. The bilateral gastrocnemius muscles together with the left regenerated nerve (at least 1 cm) were rapidly removed. Regenerated nerves (n = 3 for autograft and collagen/PCL NCs group respectively) were fixed in 4% paraformaldehyde at room temperature for 1 day for use in histology and immunohistochemistry examination. Middle portion of the regenerated nerves from another three animals in each group (n = 3 for autograft and collagen/PCL NCs group respectively) were fixed in 2% glutaraldehyde at room temperature for 1 day for use in toluidine blue staining and transmission electron microscopy. Normal nerve specimens from animals without lesions were also taken for related examinations. Gastrocnemius muscles from both sides were taken and weighed on an analytical balance immediately removed from animals. Then, middle part of the muscles were cut down and put in 4% paraformaldehyde solution for further histological examination. All the above muscle harvest procedures on animals (n = 6 for normal nerve, autograft and collagen/PCL NCs group respectively and n = 3 for non-grafted group) were completed by the same person.

#### Nerve histology and immunohistochemistry

Specimens of paraformaldehyde-fixed nerves were paraffin embedded and longitudinally sectioned. Briefly, nerve sections were incubated with rabbit anti-neurofilament 200 antibody (Sigma, USA; 1:80 diluted in PBS) at room temperature for 1 h. After washed with PBS for three times, they were further reacted with horseradish peroxidase (HRP) conjugated goat anti rabbit polyclonal antibody (Invitrogen, USA; 1:1000 diluted in PBS) at room temperature for 30 min. Through steps of coloration of 3,3'-diaminobenzidine (DAB), positive area presented as yellow dark color. Specimens without primary antibody incubation were treated as blank controls while normal nerve specimens were stained as positive controls. Meanwhile, another section of the same specimen was stained with haematoxylin-eosin (HE). Images of middle parts of the regenerated nerves were acquired under a light microscope (Olympus, Model DP72, Japan) and presented for both HE staining and anti-neurofilament staining.

#### Toluidine blue staining and transmission electron microscopy

Specimens of glutaraldehyde-fixed nerves were further post-fixed in 1% PBS-buffered osmium tetroxide at 4°C for 2 h, subsequently dehydrated through degraded ethanol solutions, infiltrated and embedded in epoxy resin transversely. Semithin sections of 500 nm thickness were stained with toluidine blue solution for light microscopy. Images were acquired under oil lens by a light microscope (Olympus, Model DP72, Japan), and measured by the image analysis program Image-Pro Plus 6.0 (Media Cybernetics, USA). Randomly selected images of a specimen (n = 3) for each group were analyzed. Myelinated nerve fiber density was calculated by myelinated nerve fiber counts in the acquired image compared to the image area. Parameter of mean diameter of regenerated myelinated nerve fibers was got by average of all inner diameter measurements (without myelin sheath thickness). Ultrathin sections of 50 nm thickness were prepared using an ultramicrotome, placed on copper grids, stained with lead citrate and examined by transmission electron microscopy (CM-120, Philip, the Netherlands).

#### Gastrocnemius muscle histology and muscle weight ratio

Specimens of paraformaldehyde-fixed gastrocnemius muscles were transversely paraffin embedded, sectioned and stained with haematoxylin-eosin (HE). Images were obtained under a light microscope (Olympus, Model DP72, Japan). Data of muscle weight was presented as a ratio of the left (experimental) side muscle weight compared to that of the right (contralateral unoperated) side. For normal nerve group, the ratio of muscle weight was defined as 1.

#### Statistical analysis

Data was presented as mean ± standard deviation where appropriate and analyzed by using a PASW Statistics 18 software package (SPSS Inc., Chicago, IL, USA). The differences between electrospun collagen/PCL and PCL meshes for analyses of fiber diameters, water contact angle and tensile properties were evaluated by Student's t test, as well as for the data of MTS assay. Multiple *in vivo *experimental groups were compared with one-way analysis of variance (ANOVA) if conditions of normality and equal variance were met. If the ANOVA returned a statistically significant p value, an LSD test was used to recognize significant differences among every two groups. Data of mean diameter of myelinated nerve fibers was tested as not equal variance, so groups were compared with nonparametric tests (Kruskal Wallis test and Mann-Whitney test). Differences were considered significant if p < 0.05.

## Authors' contributions

ZZ, WZ, XJ conceived of the study and designed the experiments. WY fabricated and characterized the composite fibrous scaffold, and performed the statistical analysis. WY, XZ, DY carried out Schwann cell culture and characterization. CZ, WZ, WY performed animal surgeries. WY, CZ, YZ conducted assessment tests of nerve regeneration and data collection. WY drafted the manuscript and XJ helped to revise it critically. All authors have read and approved the final manuscript.
